# Islet-autoantibodies in gestational diabetes and risk of progression to postpartum diabetes: a systematic review and meta-analysis

**DOI:** 10.3389/fendo.2026.1874930

**Published:** 2026-08-06

**Authors:** Arantxa González-Saitz, Susanne Kaser

**Affiliations:** Department of Internal Medicine I, Medical University of Innsbruck, Innsbruck, Austria

**Keywords:** antibodies, diabetes mellitus, gestational diabetes, meta-analysis, postpartum diabetes, pregnancy diabetes, systematic review

## Abstract

**Introduction:**

Gestational diabetes (GDM) is not only associated with markedly increased type 2 diabetes risk but is also associated with increased type 1 diabetes risk in later life. The aim of this work was to systematically evaluate the prevalence and clinical relevance of islet autoantibodies in patients with gestational diabetes.

**Method:**

A systematic literature search of the predefined keywords “gestational diabetes” or “pregnancy diabetes” and “autoantibodies” in the electronic databases PubMed, ScienceDirect, and ResearchGate from the beginning of the database until November 30, 2025 was performed. The selection of publications was based on the review of studies using predefined inclusion and exclusion criteria. The extracted data obtained were analyzed using meta-analytical techniques.

**Results:**

Twenty-one studies including a total of 7760 patients with gestational diabetes were included. Heterogeneity among the analyzed studies was high. The overall prevalence of islet autoantibodies was 10.6% (95% CI 8,2–13,0%) (I^2^ = 95,9%) in patients with gestational diabetes, which was more than seven-fold higher than in pregnant women without gestational diabetes. Prevalence was highest for ICA and anti-GAD autoantibodies. The prevalence of islet autoantibodies decreased from 10.6% during pregnancy to 3.6% in the postpartum period (95% CI 2.1–5.1%).

**Conclusion:**

Our data show a high prevalence of islet autoantibody positivity in patients with gestational diabetes suggesting a relevant pathophysiological role of autoimmunity in a subgroup of affected patients. These data underline the need of postpartum analysis of the autoantibody status and regular follow-up checks in patients with persistent islet autoantibody positivity. Heterogeneity among analyzed studies was high underlining the need of further prospective studies to more clearly define the role of islet autoantibodies in patients with gestational diabetes.

## 1 Introduction

Diabetes mellitus is one of the fastest-growing diseases worldwide. The number of people living with diabetes increased by 630 million between 1990 and 2022. Gestational diabetes mellitus (GDM) affects approximately 21 million women annually, corresponding to one in six pregnancies (1). GDM is a disorder of glucose metabolism diagnosed during pregnancy. It is primarily characterized by progressive, hormonally mediated insulin resistance, which exceeds the compensatory secretory capacity of pancreatic beta-cells and leads to elevated blood glucose levels (2, 3). Both women with a history of GDM and their offspring have an increased risk of developing type 2 diabetes mellitus later in life (2, 4). Importantly, risk of type 1 diabetes is also significantly increased in women with a history of GDM (5).

The pathogenesis of GDM is influenced by multiple factors including insulin resistance and immunological mechanisms. The autoimmune component is reflected by the presence of islet cell-specific autoantibodies in a relevant subgroup of patients (6). There are five main autoantibodies against pancreatic beta-cell antigens: autoantibodies against the 65 kDa isoform of glutamic acid decarboxylase (GAD65), autoantibodies against insulinoma-associated antigen 2 (IA-2), autoantibodies against insulin (IAA), autoantibodies against various islet cell antigens (ICA), and autoantibodies against the zinc transporter 8 (ZnT8) (7).

The detection of these autoantibodies has prompted some groups to even suggest a subdivision of GDM into an autoimmune and a non-autoimmune subgroup (7, 8). Overall, prospective studies have shown that women with GDM and a positive autoantibody profile, especially those with multiple autoantibody positivity, have a significantly increased risk of postpartum development of type 1 diabetes mellitus (9, 10).

Type 1 diabetes risk varies strongly according to age and autoantibody type with markedly lower risk in adults than in children (11). To make it even more complicated, inverse islet autoantibody conversions are frequent in children with single islet autoantibody positivity (12) and even reversions of multi-autoantibody-positivity have been described in a small subset of affected subjects (13). Importantly, islet autoantibodies are also commonly found in patients with type 2 diabetes and even in a small subgroup of healthy individuals (14–16).

As disease modifying therapies for type 1 diabetes have only recently become available, several islet autoantibody screening initiatives in children and adults were started aiming to reduce to risk of diabetic ketoacidosis and delay disease progression (17). In this study we aimed to analyze the prevalence and clinical consequences of the presence of islet autoantibodies in patients with gestational diabetes mellitus.

## 2 Materials and methods

This systematic review was designed to determine the prevalence of autoantibodies in patients with and without GDM and the progression to diabetes mellitus (type 1 and 2), and pathogenesis. If a numerical synthesis was possible, a meta-analysis was conducted.

The protocol was carried out in accordance with the guidelines of the MOOSE group (Meta-Analysis of Observational Studies in Epidemiology).

### 2.1 Data sources and search strategies

The search was conducted using electronic databases, including PubMed, ScienceDirect and ResearchGate, from the inception date of each database until November 30, 2025. The search strategy was built around controlled vocabulary and free-text terms associated with GDM and autoantibodies. The primary Boolean search string for all databases was: (gestational diabetes OR pregnancy diabetes) AND (autoantibodies).

To increase sensitivity, additional keywords were used as necessary for searching, such as: gestational diabetes, diabetes mellitus, pregnancy diabetes, postpartum diabetes, antibodies, autoantibodies, systematic review, meta-analysis. Appropriate adaptations in syntax were made to the search terms to match each database. In the PubMed database, a combination of Medical Subject Headings (MeSH) terms and keywords were used. ScienceDirect was searched in title, abstract and keyword fields. The ResearchGate database was not used as a primary bibliographic database but rather as a tool to discover potentially relevant articles, conference presentations, author-shared documents, and research studies that may not be accessible from conventional database searches. All records found in ResearchGate were checked for eligibility and reviewed against this article through full-text review in the same manner as for the studies found in PubMed and ScienceDirect databases. No language restriction was imposed during this search.

All identified documents from the initial database were reviewed at the title and abstract levels.

This initial screen was followed by full-text review of potentially relevant studies according to predefined inclusion criteria. Focus was also placed on confirming the scientific legitimacy of every publication. Only legitimate, published peer-reviewed studies that met the requirements outlined above, and had adequate methodological rigor were included in the analysis:

OpenEvidence was also used as an AI-supported tool for literature research, which is applied in evidence-based research.

### 2.2 Inclusion and exclusion criteria

Studies were included for analysis if they met the following criteria: 1) the subjects were pregnant women with GDM using criteria that are recognized in the medical community, 2) prevalence or frequency of one or more diabetes-associated autoantibodies were measured in these pregnant women, 3) relevant data that were sufficient for extraction and analysis were presented, and 4) the study design was either prospective or retrospective and observational (cohort, case–control, and cross-sectional). If comparative analyses between GDM and the general population were performed, the studies were included in this comparison if an appropriate control group of subjects without GDM were provided. If the authors reported any outcomes measuring the clinical significance of positive anti–insulin autoantibody status, including, but not limited to, C-peptide levels, intra-uterine insulin demand, oral glucose tolerance test results, postpartum plasma glucose concentration, and conversion to type 1 and type 2 diabetes after pregnancy these studies were also included. We did not place any restrictions on publication date, location, or language.

We reviewed and extracted data from studies in any language if the information was sufficient. We excluded studies if: 1) the patients had diagnosed Type 1 and Type 2 Diabetes Mellitus prior to the pregnancy, 2) non-original work including reviews, letters, opinion pieces, commentaries, editorials, conference abstracts where key data are not reported, and case series; 3) no data was provided regarding diabetes related autoantibodies in GDM population; 4) lack of sufficiently complete information for analysis of any of the study variables; 5) overlapping of participant populations and results, in which case the most comprehensive recent study published will be selected. All identified potentially suitable articles were screened at title and abstract level and thereafter those that met criteria were critically assessed at full paper level. Only articles that fulfilled all inclusion criteria and none of the exclusion criteria will be included in the qualitative and quantitative synthesis.

### 2.3 Selection methods

**Figure 1:**
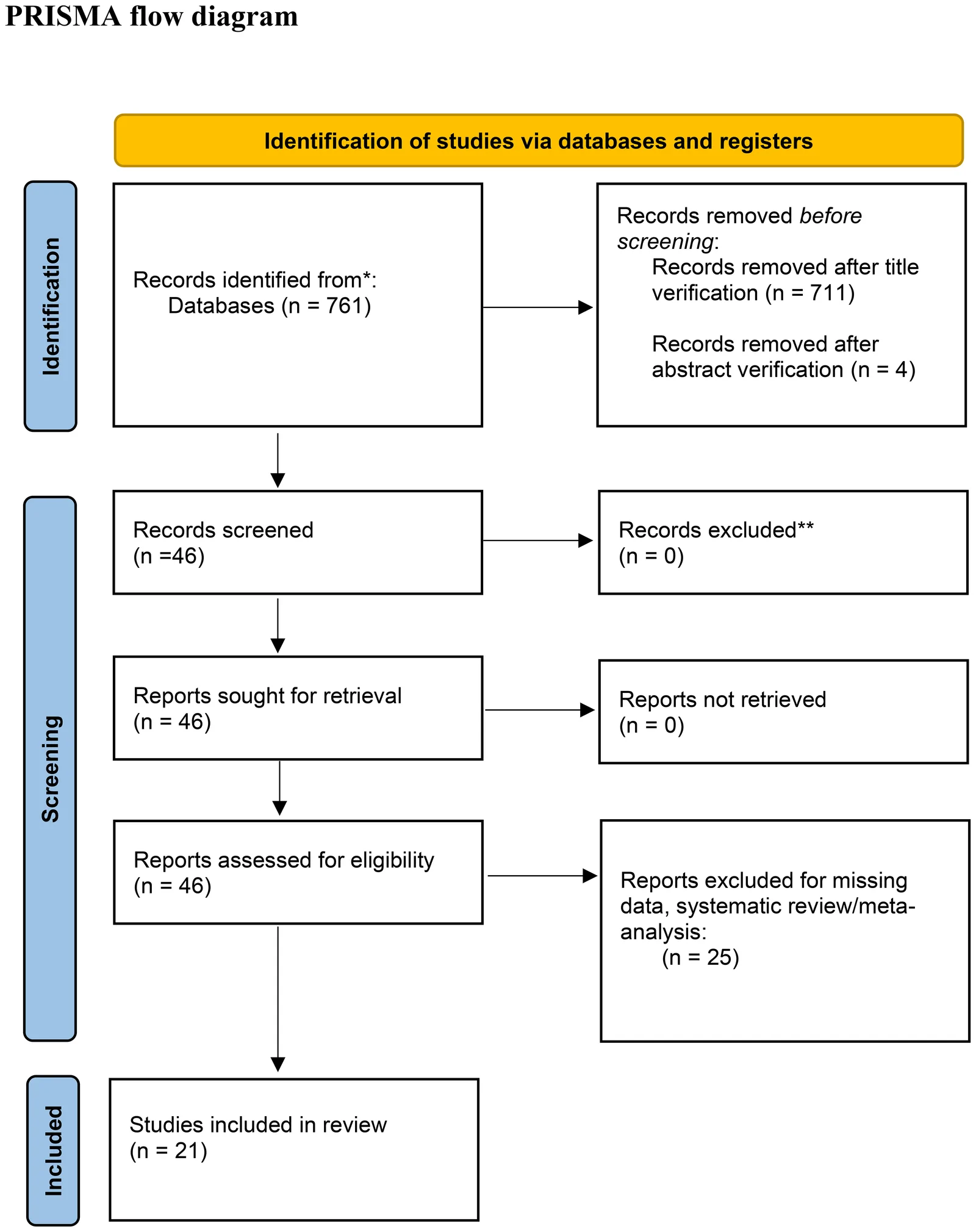
A simplified PRISMA flow diagram of methodology. PRISMA, Preferred Reporting Items for Systematic Reviews and Meta-Analyses. PageMJ, McKenzie JE, Bossuyt PM, Boutron I, Hoffmann TC, Mulrow CD, et al. The PRISMA 2020 an updated guideline for reporting systematic reviews BMJ 2021; 372:n71 doi: 10.1136/bmj.n71. Open License, Prisma-Satement.org, material has been adapted). *Pubmed, ScienceDirect and ResearchGate. **No automation tools were used; all records were excluded by a human.

The author of the study and the supervisor independently reviewed the articles and resolved any discrepancies by consensus. First, duplicate articles were removed. Then, publications were selected primarily based on title screening, followed by abstract review. After an initial screening, the full text of the studies was examined (**Figure 1**).

### 2.4 Data extraction

The following data were extracted from each included study: first author, year of publication and country, number of GDM patients and control group, antibody findings, and results with predictive value for the further development of diabetes mellitus.

### 2.5 Quality assessment

The quality of the included studies was assessed using the Quality Assessment Tool for Observational Cohort and Cross-Sectional Studies (National Heart, Lung, and Blood Institute at the National Institutes of Health, USA), available at https://www.nhlbi.nih.gov/health-topics/study-quality-assessment-tools. It includes 14 criteria addressing objectives, population, proportion of eligible participants, sample size, exposure, outcomes, blinded assessors, follow-up, and confounding variables.

Quality Assessment revealed that of the 21 studies included in the systematic review, 14 were rated as good and 7 as fair quality. The lack of any studies deemed of poor quality suggests an overall moderate to high level of methodological quality for the included studies. The larger number of studies rated as good quality increases confidence in the pooled estimates because it means that major sources of bias, such as inappropriate participant selection, poor outcome ascertainment and lack of adequate control of confounders are less likely to have significantly impacted the results.

In studies rated fair quality, the researchers identified a methodological limitation such as the participants in a proportion of studies had some losses to follow-up, confounding was adjusted for with limited precision, or only partial details were provided for the design and implementation of the study.

Nevertheless, studies rated fair quality were included in the review since they meet inclusion criteria and provide relevant data. Limitations in these studies are noted when interpreting the study findings. The estimates derived from this meta-analysis, therefore, must be interpreted with consideration for the various quality levels, giving precedence to the consistency of findings in the good quality studies.

### 2.6 Statistical data analysis

Two freely available programs were used to perform the meta-analysis: ReviewManager, a customized software from Cochrane Training, and OpenMeta[Analyst], an open-source software from Brown University (USA). The weighted prevalence of autoantibodies in GDM was calculated using the DerSimonian-Laird random-effects model, and the results are presented together with the 95% confidence interval (95% CI). To compare the prevalence of antibodies between patients with GDM and controls, a meta-analysis using the Mantel-Haenszel random-effects model was performed. The results are presented as odds ratios with 95% CI. Heterogeneity between studies was assessed using the I² statistic. Publication bias was evaluated using a funnel plot. In all cases, the confidence level was set at 95% (p < 0.05).

## 3 Results

The initial search strategy yielded 761 records. After removing duplicate studies that were not relevant or eligible to our research objective, 46 articles of interest remained. Of these, 25 were excluded for the following reasons: missing data, systematic reviews, or meta-analyses. Ultimately, a total of 21 studies reporting original data were included (5, 18–37). The population-based study by Papadopoulou et al. (38) risks partial patient overlap with another prospective trial of the research group (23) why it was not included in the meta-analysis. The characteristics of included studies are presented in **Tables 1**–**3**. The studies used for the statistical analysis were published between 1990 and 2023. They were conducted in twelve different countries: Australia, Belgium, Denmark, Germany, Finland, India, Italy, Sweden, Spain, South Korea, the USA, and the United Kingdom. The studies were divided into two groups depending on the presence of a control group.

**Table 1:** Characteristics of included studies with control group assessing islet autoantibodies in women with GDM.

Study (first author, year)	Country	GDM participants (n)	Controls (n)	Autoantibodies assessed	Outcome(s) evaluated
P. M. Catalano (1990) (18)	United States	187	5	ICA	Prevalence OGTT outcomes
R. C. McEvoy (1991) (19)	United States	144	108	Anti-GAD, IAA, ICA	Autoantibody prevalence
J. S. Petersen (1996) (20)	Denmark	139	27	Anti-GAD	Autoantibody prevalence; clinical outcomes
M. Balaji (2002) (21)	India	86	114	Anti-GAD, Anti-IA2	Autoantibody prevalence
I. Järvelä (2006) (5)	Finland	395	388	Anti-GAD, Anti-IA2, IAA, ICA	Autoantibody prevalence; clinical outcomes
C. Murgia (2008) (22)	Italy	62	56	Anti-GAD, Anti-IA2, IAA, ICA	Autoantibody prevalence
A. Papadopoulou (2012) (23)	Sweden	418	138	Anti-GAD, Anti-IA2	Autoantibody prevalence; clinical outcomes
E. Cossu (2018) (24)	Italy	143	60	Anti-GAD, Anti-IA2, IAA, ZnT8-A	Autoantibody prevalence
K. Luiro (2023) (25)	Finland	342	353	Anti-GAD, Anti-IA2, IAA, ICA	Autoantibody prevalence; clinical outcomes

**Table 2:** Characteristics of included studies assessing islet autoantibodies in women with GDM.

Study (first author, year)	Country	GDM participants (n)	Autoantibodies assessed	Outcome(s) evaluated
D. Mauricio (1995) (26)	Spain	534	ICA	Autoantibody prevalence
N. A. Beischer (1995) (27)	Australia	734	Anti-GAD	Autoantibody prevalence; clinical outcomes
M. Füchtenbusch (1997) (28)	Germany	437	Anti-GAD, Anti-IA2, ICA	Autoantibody prevalence; clinical outcomes
J. L. Bartha (2000) (29)	Spain	235	Anti-GAD, ICA	Autoantibody prevalence; clinical outcomes
E. Kousta (2001) (30)	United Kingdom	321	Anti-GAD	Autoantibody prevalence
K. Löbner (2006) (31)	Germany	302	Anti-GAD, Anti-IA2	Autoantibody prevalence; clinical outcomes
C. Nilsson (2007) (32)	Sweden	385	Anti-GAD, Anti-IA2, ICA	Autoantibody prevalence; clinical outcomes
Y. Sung Hoon and P. Sunmin (2009) (33)	South Korea	887	Anti-GAD	Autoantibody prevalence; clinical outcomes; OGTT outcomes
J. Dereke (2012) (34)	Sweden	193	Anti-GAD, Anti-IA2, IAA, ZnT8-A	Autoantibody prevalence; clinical outcomes
T. P. Lundberg (2015) (35)	Denmark	407	Anti-GAD	Autoantibody prevalence; clinical outcomes
J. Dereke (2016) (36)	Sweden	1225	Anti-GAD	Autoantibody prevalence; clinical outcomes
K. Beunen (2022) (37)	Belgium	186	Anti-GAD, Anti-IA2, IAA, ICA	Autoantibody prevalence; OGTT outcomes

**Table 3:** Clinical outcomes of autoantibody-positive women with GDM.

Study (first author, year)	Follow-up period	Autoantibody prevalence	Progression to type 1 diabetes mellitus (T1DM)	Key findings
P. M. Catalano (1990) (18)	Cross-sectional	1.6%	Not reported	ICA positivity was associated with reduced first-phase insulin secretion.
N. A. Beischer (1995) (27)	Variable postpartum follow-up	1.8% in GDM; 100% in insulin-deficient diabetes; 7.1% in non–insulin-dependent diabetes	Variable	GADA identified a subgroup with insulin-dependent diabetes.
M. Füchtenbusch (1997) (28)	Up to 7 years	18.1%	29% (Ab+) vs. 2% (Ab−) after 2 years	The number of autoantibodies was strongly predictive of T1DM risk; women with three antibodies had an 84% risk of progression.
J. L. Bartha (2000)	Postpartum	13.7% of women with early GDM were GADA-positive versus 9.3% with late GDM; one woman was ICA-positive	Higher among women with early-onset GDM	Early GDM onset was a stronger predictor than antibody status; postpartum diabetes occurred in 26.7% and glucose intolerance in 40% of participants.
E. Kousta (2001) (30)	Median 22 months postpartum	4%	Not reported	No ethnic differences were observed; GADA-positive women had lower BMI.
K. Löbner (2006) (31)	5.7 years	Among T1DM cases: ICA 67%, GADA 56%, IA-2A 38%	4.6% developed T1DM; 5.3% developed type 2 diabetes mellitus (T2DM)	Autoantibodies were predominantly detected in women who developed T1DM and were uncommon in T2DM.
I. Järvelä (2006) (5)	5.7 years	ICA detected in 67% of women who developed T1DM	4.6% developed T1DM	Age ≤30 years, insulin treatment during pregnancy, and autoantibody positivity predicted progression to T1DM.
A. Papadopoulou (2012) (23)	1–2 years postpartum	Not explicitly reported	Not directly reported	GADA positivity was associated with postpartum diabetes; HLA-DQB1*0602 was negatively associated with diabetes development (p = 0.017).
T. P. Lundberg (2015) (35)	3 months postpartum	5.4%	Not a primary endpoint	GADA-positive women had lower C-peptide concentrations and a reduced disposition index.
K. Beunen (2022) (37)	4.6 years	8.1%	One case of T1DM reported	Persistence of autoantibodies was observed; repeat antibody testing was recommended.
K. Luiro (2023) (25)	23 years	17%	100% among women with ≥3 autoantibodies (within 7 years)	This study provided the longest follow-up; multiple autoantibodies were highly predictive of future T1DM.

### 3.1 Prevalence of islet autoantibodies in GDM

**Figure 2:**
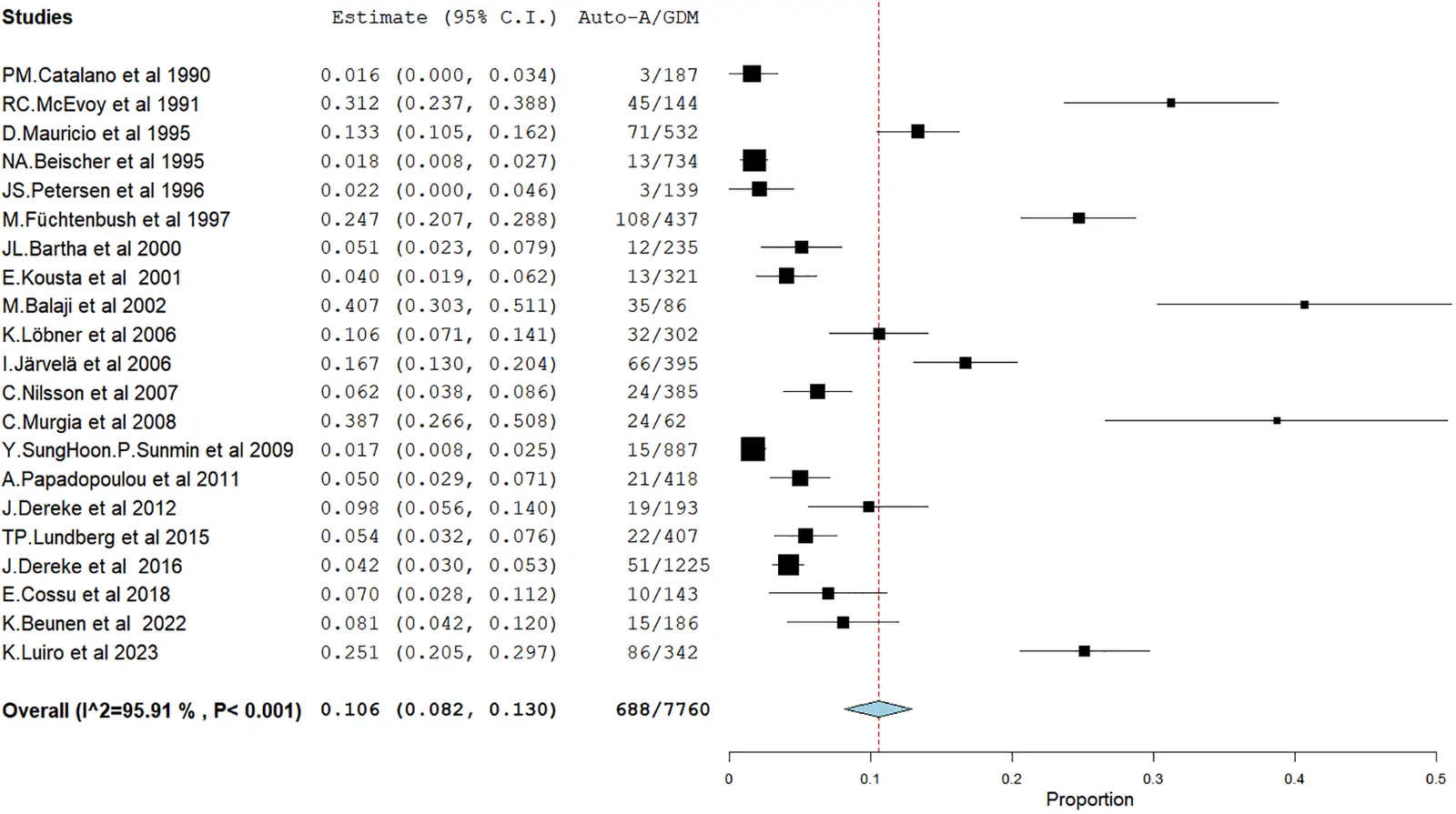
Prevalence of islet autoantibodies positivity in patients with GDM. Forest plot of prevalence of islet autoantibody positivity in pregnant women with GDM is shown. 21 studies including 7760 patients were analyzed (5, 18–37).

**Figure 2** shows the forest plot of the prevalence of islet autoantibodies in patients with GDM. Twenty-one studies including 7760 pregnant women with GDM (5, 18–37) were analysed. Heterogeneity of analyzed studies was very high (I2 = 95,9**%**). The average prevalence was 10.6% (95% CI 8.2–13.0%).

**Figure 3:**
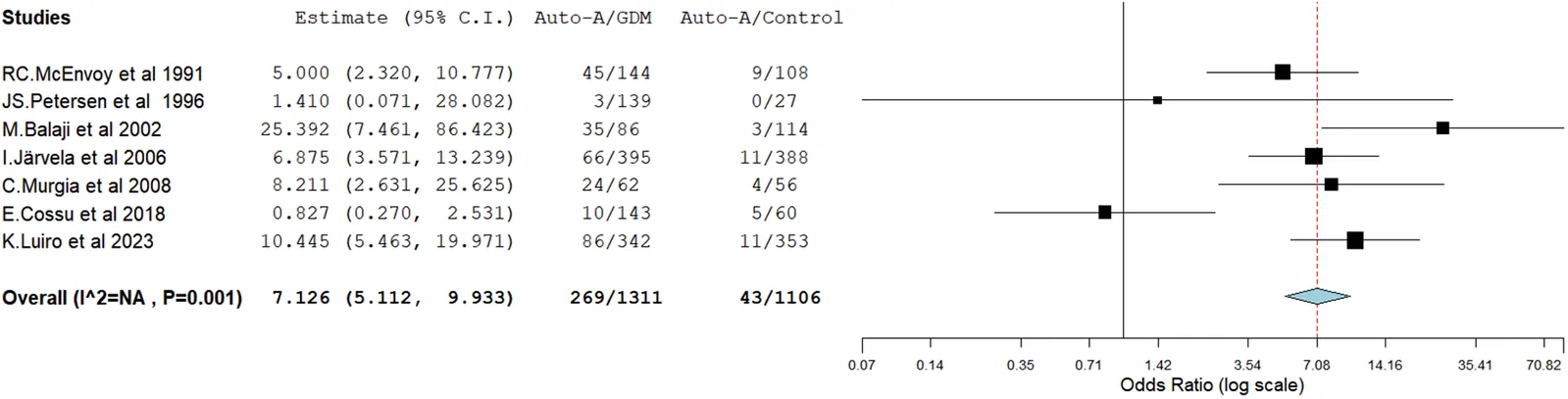
Prevalence of islet autoantibody positivity in pregnant women with and without GDM. Forest plot comparing prevalence of islet autoantibody positivity in patients with GDM (n=1311) and pregnant women without GDM (n=1106) is shown (5, 19–22, 24, 25).

Of these 21 mentioned studies, a total of 7 included a control group. Altogether, they comprised 1311 women with GDM and 1106 pregnant women without GDM (5, 19–22, 24, 25). The presence of islet autoantibodies was more than 7fold higher in women with GDM than in those without GDM (OR 7.126 [5.112, 9.933], p < 0.001) (**Figure 3**).

### 3.2 Prevalence of islet autoantibodies after GDM

**Figure 4:**
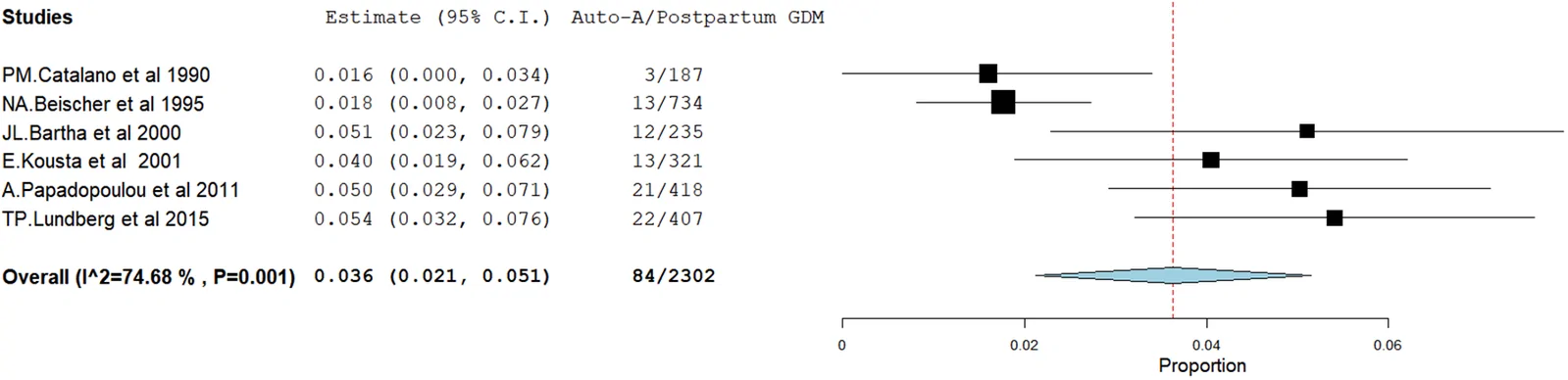
Prevalence of islet autoantibody positivity in the postpartum period. Forest plot of prevalence of islet autoantibody positivity is shown. 6 studies including 2302 patients with GDM were analyzed. Laboratory analyses were performed 1 week to 22 months after delivery (18, 23, 27, 29, 30, 35).

**Figure 4** shows the forest plot of the prevalence of autoantibodies after GDM (antibody measurements ranged from 1 week postpartum to 22 months): In total 6 studies including 2,302 patients with GDM (18, 23, 27, 29, 30, 35) were analyzed. In one study (18) prevalence data were only available for the postpartum period, while in the other studies analyses were performed during pregnancy and in the postpartum period. The overall prevalence was 3.6% (95% CI 2.1–5.1%) with a heterogeneity of I^2^ 74,68%. In the study by Papadopoulou et al. (38) which was excluded from meta-analysis due to the risk of partial overlap with another included study (23), 7.6% of patients with GDM were found positive for at least of one islet cell autoantibody (GAD, IA-2, IA antibodies).

### 3.3 Prevalence of the different types of islet autoantibodies in GDM

**Figure 5:**
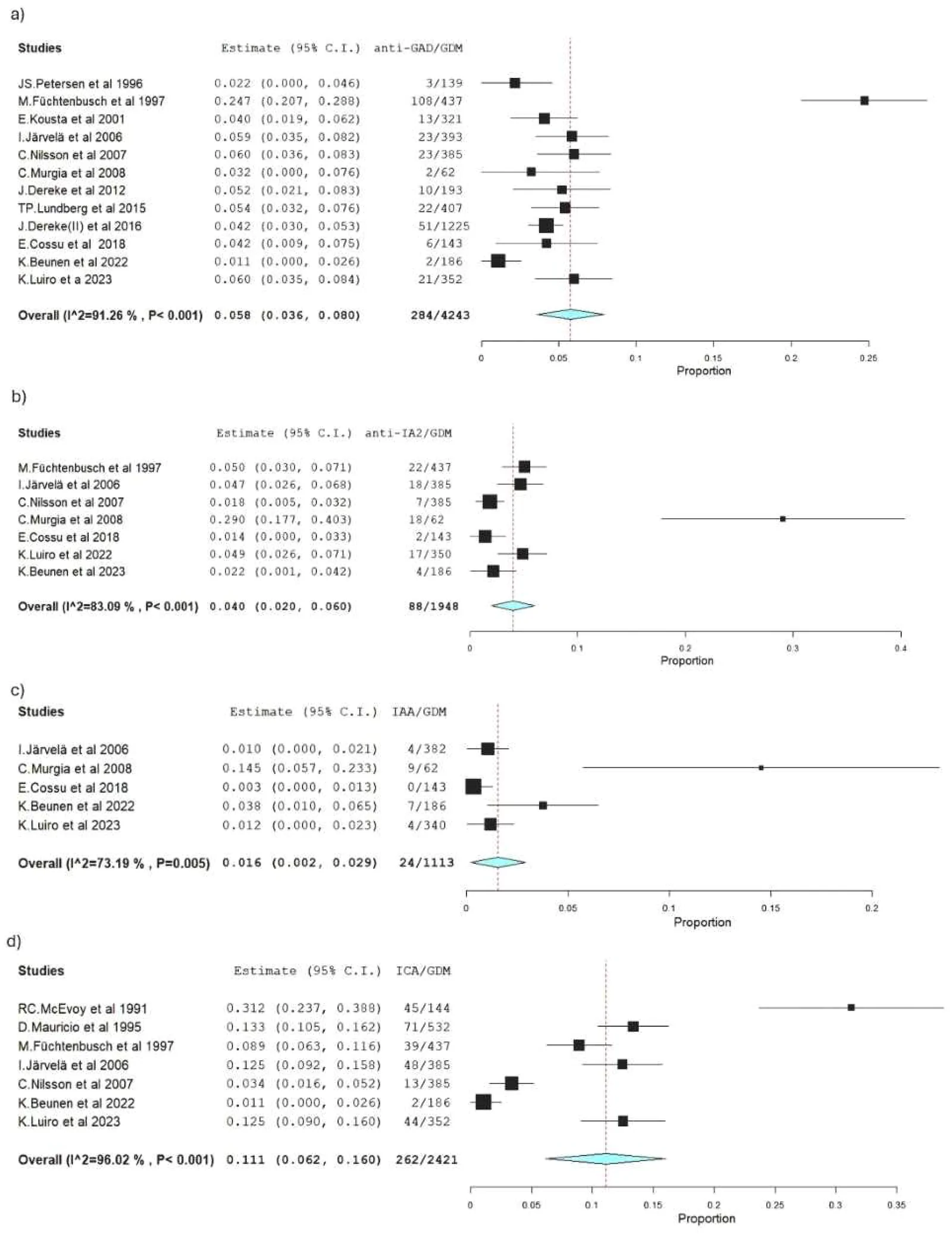
Prevalence of specific islet autoantibody positivity in patients with GDM. Forest plots for anti-GAD **(a)**, anti-IA-2 **(b)**, IAA **(c)** and ICA **(d)** autoantibody positivity in patients with GDM are shown. **(a)** Anti-GAD was available in 12 studies including a total of 4243 patients with GDM (5, 20, 22, 24, 25, 28, 30, 32, 34–37). The prevalence was 5.8% (95% CI 3.6–8.0%). **(b)** Anti-IA2 autoantibody positivity was reported in 7 studies analyzing a total of 1948 patients with GDM (5, 22, 24, 25, 28, 32, 37) with an overall prevalence of 4.0% (95% CI 2.0–6.0%). **(c)** IAA autoantibodies were available from 5 studies investigating 1113 patients with GDM (5, 22, 24, 25, 37). The prevalence was 1.6% (95% CI 0.2–2.9%). **(d)** ICA autoantibodies were available from 7 studies including a total of 2421 patients with GDM (5, 19, 25, 26, 28, 32, 37). The prevalence was 11.1% (95% CI 6.2–16.0%).

**Figure 5** shows the forest plots for anti-GAD autoantibody (a), anti-IA2 (b), anti-IAA (c) and ICA antibody (d) positivity in patients with GDM. Control groups were included only in a subgroup of studies why additional subgroup analyses have not been performed.

Anti-GAD autoantibody positivity was analyzed in 12 studies including a total of 4243 patients with GDM (5, 20, 22, 24, 25, 28, 30, 32, 34–37). Five studies included a control group (5, 20, 22, 24, 25). The prevalence of anti-GAD autoantibody positivity was 5.8% (95% CI 3.6–8.0%).

Anti-IA2 autoantibody positivity was reported in 7 studies analyzing a total of 1948 patients with GDM (5, 22, 24, 25, 28, 32, 37) with an overall prevalence of 4.0% (95% CI 2.0–6.0%). Four studies included a control group (5, 22, 24, 25). Data on anti-IAA autoantibodies were available from 5 studies investigating 1113 patients with GDM (5, 22, 24, 25, 37) including 4 studies with control groups (5, 22, 24, 25). Overall prevalence of anti-IAA autoantibody positivity was low (1.6% (95% CI 0.2–2.9%)).

Anti-ICA autoantibody positivity was more frequently than anti-GAD, anti-IA2 or anti-IAA autoantibody positivity, respectively, with an overall prevalence of 11.1% (95% CI 6.2–16.0%). Data were available from 7 studies including a total of 2421 patients with GDM (5, 19, 25, 26, 28, 32, 37). Only 3 studies included control groups (5, 19, 25). Importantly, ICA prevalence was calculated only among patients who were tested for ICA, whereas the overall prevalence represents a weighted estimate across all studies. In this case, larger cohorts were included, which therefore showed a lower antibody prevalence. The two prevalence estimates are not directly comparable but do not represent a statistical contradiction. As performance of ICA autoantibody assays has dramatically evolved over time, a subgroup analysis was performed including studies published between 2006 and 2023 only. In this subgroup analysis anti-ICA autoantibody positivity was found in 7.1% (95% CI: 1.9%–12.4%) (**Supplementary Figure 1**).

In the study by Papadopoulou et al. (38) prevalences for GAD, IA-2 and IA antibodies were 4.3, 1.3 and 3.2%, respectively.

### 3.4 Persistence of islet autoantibodies and inverse seroconversion

Overall, only few data were available on the natural course of islet autoantibody positivity postpartum. Füchtenbusch et al. (1997) reported different persistence rates depending on the type of autoantibody: GADA persisted in 75% of positive patients, whereas ICA persisted in only 35% and IA-2A in 30% (21).

Lapolla et al. (2002) reported that 2 out of 5 with gestational diabetes mellitus with single autoantibody positivity maintained seropositive in the postpartum period (39). In a Belgian study Beunen et al. (2022) confirmed that autoantibodies can persist for years after pregnancy; 4 out of 12 women remained positive after 4.6 years (37).

### 3.5 Predictive value for the development of type 1 diabetes

Only few studies with small sample sizes were available investigating the predictive value of islet autoantibody positivity in patients with GDM: Füchtenbüsch et al. reported that the number of positive autoantibodies is strongly associated with type 1 diabetes risk in the long term. Patients with a single autoantibody positivity had a 17% risk of developing type 1 diabetes within two years; with two islet autoantibodies found positive, the risk increased to 61%, and in patients with three positive islet antibodies the risk was 84% (28).

This finding is further supported by the 23-year Finnish follow-up study by Luiro et al., which showed that all GDM patients with three positive autoantibodies developed type 1 diabetes within seven years after the GDM pregnancy (33). In this study, positivity for anti-ICA, anti-GAD and/or anti IA-2 autoantibody but not anti-IAA autoantibody positivity.

### 3.6 Metabolic correlates: C-peptide and beta-cell function

Catalano et al. was the first to show that ICA autoantibody-positive patients with GDM display a reduced early-phase insulin response, indicating progressive damage to pancreatic islet cells (18). This observation was expanded by Lundberg et al. who demonstrated that fasting C-peptide levels (520 vs 761 pmol/L, p=0.02) and disposition índices were significantly lower in GAD-autoantibody positive patients with GDM than in patients with a negative autoantibody status (35).

The timing of GDM diagnosis might provide additional prognostic information independent of autoantibody status. Bartha et al. (2001) compared 30 women diagnosed with GDM early in pregnancy with 72 women who were diagnosed later in pregnancy (29). Women with early-onset GDM showed significantly higher rates of postpartum metabolic dysfunction: overt diabetes (26.7% vs. 1.4%, p = 0.0002) and impaired glucose tolerance (40% vs. 5.56%, p < 0.0001).

Interestingly, the rate of GAD antibody positivity was similar in both groups (13.7% vs. 9.3%) (29).

## 4 Discussion

Only recently, disease-modifying therapies for patients at high risk of type 1 diabetes have become available. As islet-specific autoantibodies strongly predict type 1 diabetes risk (40), huge national and international screening programs have been launched for identification of high-risk patients.

Gestational diabetes mellitus is a well-known and strong risk factor for type 2 diabetes in later life. In a recent meta-analysis Vounzoulaki et al. (41) found that type 2 diabetes risk is about 10fold higher in patients with a history of GDM than in those with a history of normoglycemic pregnancy. In another meta-analysis, Dennison RA et al. found that one third of women with a history of GDM are prone to develop type 2 diabetes within 15 years (42). Very interestingly, type 2 diabetes conversion risk was especially high in parts of Central and South America and South Africa, while risk was significantly lower in White European populations.

While the high type 2 diabetes risk in patients with GDM is well established, type 1 diabetes risk was also found to be markedly elevated in patients with GDM (5). In a prospective study, 4.6% of patients with a history of GDM developed type 1 diabetes. Younger age, autoantibody positivity and insulin need during pregnancy were associated with increased type 1 diabetes risk in this study (5).

The aim of this study was to define prevalence, postpartum dynamics and metabolic consequences of islet autoantibody positivity in patients with GDM by performing a meta-analysis and systematic review.

Importantly, the number of prospective, controlled studies is very limited. Furthermore, heterogeneity of studies is very high which might be explained by several factors: first, the definition of GDM which substantially changed over time and screening procedures still vary by country, second, time points of autoantibody analysis differed greatly between the studies. Third, different assays with varying sensitivity and specificity were used for islet autoantibody analysis in the studies and antibody titers were either not shown or not comparable between the studies. Fourth, sample size of most studies was small. Additionally, ethnic differences might also contribute to huge heterogeneity of the analysis.

In our analysis we found a prevalence of 10.6% in women with GDM with the highest levels observed for the presence of anti-ICA and anti-GAD autoantibodies. Importantly, high prevalence especially of ICA autoantibodies should be interpreted with caution for methodological reasons. In the oldest study included (19) ICA autoantibodies were determined using a complement-mediated cytotoxicity assay. ICA positivity was diagnosed when more than 50% of rat insulinoma cells were killed. Noteworthy, performance of autoantibody assays has evolved dramatically over time transitioning from subjective classical fluorochroms and indirect immunofluorescence to time resolved fluorescence imaging, radiobinding assays, radioimmunoassay, enzyme linked immunosorbent assays and electrochemiluminescence assays (43–46). Despite enormous methodological improvements, discordance of results between assays remains high. In a recent study, concordant results were 79.7% for anti-GAD, 65.2% for anti IA-2, 36.7% for anti-IAA and 67.5% for ZnT8A autoantibody analysis when performing tests in 5 different laboratories (45). To assess the robustness and contemporary relevance of our findings of ICA prevalence, we performed a subgroup analysis including only studies published between 2006 and 2023. This analysis yielded a pooled prevalence of 7.1% (95% CI: 1.9%–12.4%), compared with 11.1% when all studies published between 1991 and 2023 were included. The lower prevalence observed in the more recent studies suggests that the inclusion of older studies may have contributed to an overestimation of the overall pooled prevalence. Notably, despite the exclusion of older studies, ICA remained the most prevalent autoantibody subtype, indicating that the predominance of ICA was not solely driven by earlier publications. However, substantial heterogeneity persisted (I² = 95.31%), indicating considerable variability among the included studies.

The prevalence of diabetes-associated autoantibodies in GDM is markedly elevated, being approximately 5- to 11-fold higher than in the general non-diabetic population, where islet autoantibodies are typically detected in only 1–2% of individuals (46, 47). Very importantly, prevalence of islet autoantibody positivity was more than 7-fold higher in patients with GDM than in pregnant women without a history of GDM. Noteworthy, diagnostic criteria of GDM varied greatly between the studies and ADA 2017/IADPSG (48, 49) diagnostic criteria were only used in 2 studies (24, 37). In contrast, in the study by Luiro et al. (25) GDM was diagnosed by insulin treatment or oral glucose test according to the Finnish Diabetes Association (fasting, ≥4.8 mmol/L; 1-hour, ≥10.0 mmol/L; and 2-hour, ≥8.7 mmol/L), however, GDM screening via oral glucose tolerance tests were performed in high-risk patients only. Accordingly, great variations in the prevalence of islet autoantibody positivity in these recent studies might not only be explained by differences in ethnical background but also by differences in diagnostic criteria and screening procedures. In summary, despite a huge heterogeneity our analysis suggests that a relevant subgroup of patients with GDM test positive for islet autoantibodies.

Multiple positive autoantibodies were found to correlate with an increased risk of postpartum progression to type 1 diabetes within seven years in a previous study (25). Islet autoantibody-positive patients with GDM were reported to commonly have genetic risk factors such as HLA-DR3 or DR4 alleles and also exhibit a phenotype similar to patients with type 1 diabetes with low to normal body mass index before pregnancy, preserved insulin sensitivity, and a higher likelihood of requiring insulin therapy during pregnancy when compared to autoantibody-negative patients with GDM (50, 51). Both, number and type of autoantibodies are prognostically relevant as autoantibody multi-positivity was associated with dramatically increased type 1 diabetes risk after delivery in several studies (9, 25, 46, 51). These findings suggest that autoantibody-positive GDM might represent a distinct pathophysiological entity characterized by autoimmune destruction of beta cells (52–56). We thus suggest that impaired beta-cell function should be considered in autoantibody-positive patients with GDM, especially in those at young age and who are treated with insulin (5). The timing of GDM diagnosis might also provide additional prognostic information as early-onset GDM might rather reflect the manifestation of pre-existing autoimmune or metabolic dysfunction than pregnancy-induced glucose intolerance (29).

The question of universal versus targeted autoantibody screening remains unresolved and controversial. Beunen et al. concluded that systematic screening is not justified due to low overall positivity rates but recommended repeated titer measurements in women with clinically relevant antibody levels (37). The mechanisms underlying immune modulation during and after pregnancy remains unclear. Remarkably, we found that prevalence of autoantibody positivity was significantly lower in the postpartum period than during pregnancy in patients with GDM which might be discussed as inverse seroconversion in a relevant subgroup of patients with GDM.

Pregnancy is usually associated with an immunological shift with a balance in favor of anti-inflammatory responses (57, 58) and immunotolerance of the microchimeric milieu resulting from trafficking of fatal cells into the maternal circulation (59). Very interestingly, long-term persistence of male fetal progenitor cells was found in a subgroup of mothers of sons (60). Taken together these data suggest a great individual variability in immune response in pregnancy and postpartum. While the mechanisms of inverse seroconversion remain unclear, these data indicate that autoantibody positivity in patients with gestational diabetes mellitus is often transient and does not necessarily reflect autoimmune diabetes (38). Accordingly, confirmation of autoantibody positivity is recommended in general to exclude transient immune response. For confirmation, autoantibody status should be assessed in a laboratory that meets the performance standards set by the Islet Autoantibody Standardization Program (IASP) (17, 61).

Given the high predictive value of multiple autoantibodies for the development of type 1 diabetes as shown by Luiro et al. (25) and Füchtenbusch et al. (28), screening in high-risk groups may not be only cost-effective—particularly in women with early-onset GDM, insulin requirement during pregnancy, or younger age - but may also reduce risk of life-threatening diabetic ketoacidosis. Interestingly, autoantibody positive patients displayed higher fasting and 2-hour glucose values during the oral glucose tolerance test suggesting impaired beta-cell function and reduced insulin secretion in affected women when compared to autoantibody negative patients with GDM (35). Taken together these data support tight follow-up analysis of islet autoantibodies and glycemia in the postpartum period in patients with GDM.

This review and meta-analysis includes a comprehensive literature search using three major bibliographic databases and an extensive and in-depth analysis. However, conclusions of our data might be limited by the high heterogeneity of analyzed studies. Importantly, autoantibody negative and autoantibody positive patients with GDM were matched for age between the groups. Additional limitations include the fact that not all publications provided a complete data set and that diagnostic criteria for defining islet autoantibody positivity versus autoantibody negativity varied slightly between the studies. Furthermore, a selection bias of participating patients especially in prospective studies cannot be ruled out. Potentially relevant studies published after 30^th^ November 2025 have not been included in our analysis.

In conclusion, our results show that patients with GDM have a markedly higher prevalence of diabetes-associated autoantibodies, particularly ICA and anti-GAD autoantibodies than pregnant women without GDM or healthy controls suggesting an autoimmune subgroup within the GDM population. These data underline the need of regular follow-up checks including analysis of islet autoantibody status postpartum in autoantibody positive patients with GDM. Further prospective studies are needed to determine mechanisms and rates of inverse seroconversion and the long-term type 1 diabetes risk in these patients.

## Data Availability

The original contributions presented in the study are included in the article/**Supplementary Material**, further inquiries can be directed to the corresponding author/s.

## Supplementary material

Supplementary Figure 1 Subgroup analysis of ICA autoantibody status including studies published between 2006 and 2023 only.

Supplementary Table 1 Risk-of-bias/quality assessment.

Supplementary Table 2 Characteristics of study populations and dates of autoantibody analysis in included studies with control groups.
