# Correction: Islet-autoantibodies in gestational diabetes and risk of progression to postpartum diabetes: a systematic review and meta-analysis

**DOI:** 10.3389/fendo.2026.1964758

**Published:** 2026-08-24

**Authors:** Frontiers Production Office

**Affiliations:** Frontiers Media SA, Lausanne, Switzerland

**Keywords:** antibodies, diabetes mellitus, gestational diabetes, meta-analysis, postpartum diabetes, pregnancy diabetes, systematic review

There was a mistake in the caption of **Figure 1**. The copyright citation and the labelling for * and ** were missing (see below).

**Figure 1:**
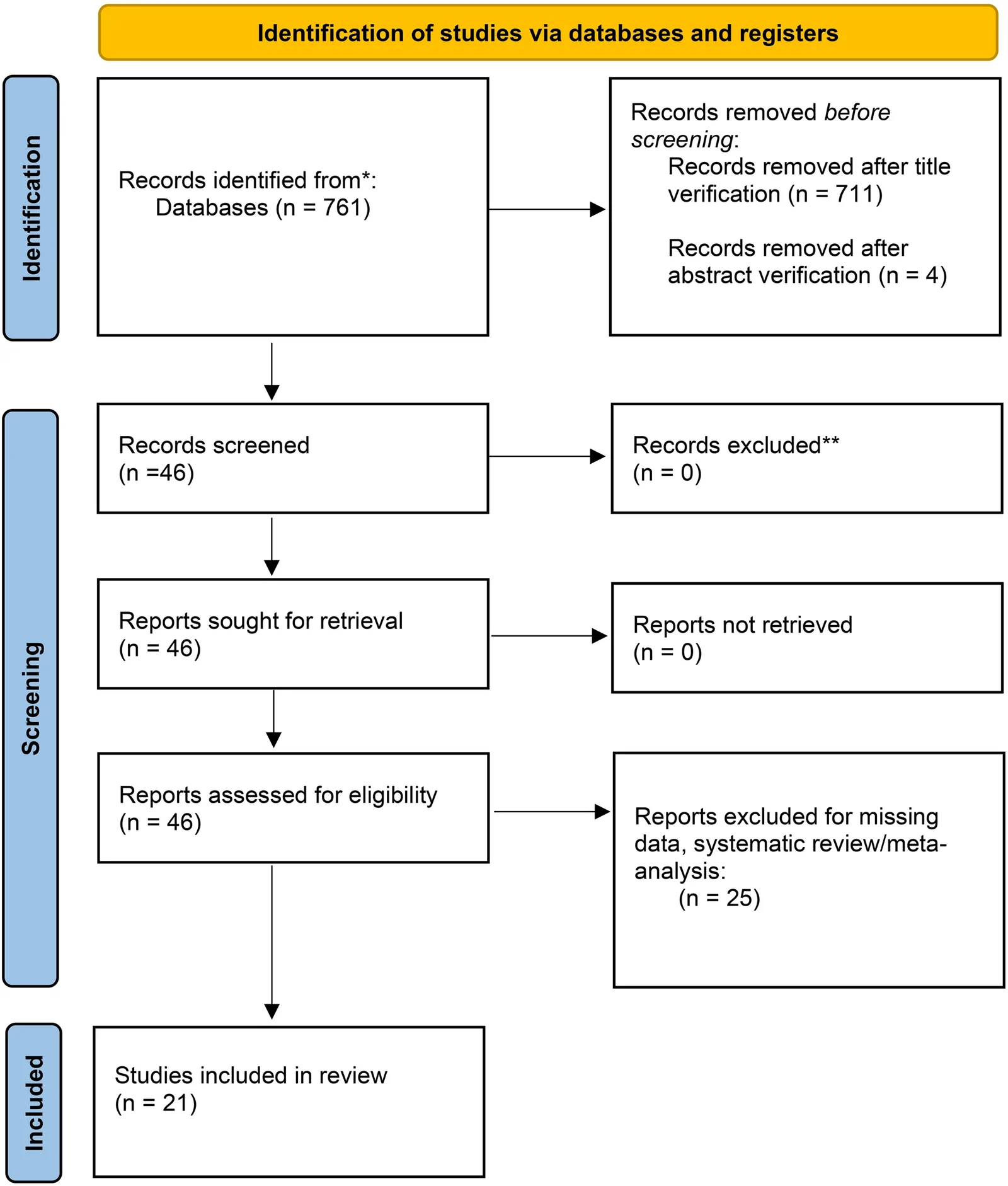
A simplified PRISMA flow diagram of methodology. PRISMA, Preferred Reporting Items for Systematic Reviews and Meta-Analyses. PageMJ, McKenzie J E, Bossuyt PM, Boutron I, Hoffmann T C, Mulrow C D et al. The PRISMA 2020 an updated guideline for reporting systematic reviews BMJ 2021; 372:n71 doi:10.1136/bmj.n71. Open License, Prisma-Satement.org, material has been adapted). *Pubmed, ScienceDirect and ResearchGate. **No automation tools were used; all records were excluded by a human.

“Page M J, McKenzie J E, Bossuyt P M, Boutron I, Hoffmann T C, Mulrow C D et al. The PRISMA 2020 an updated guideline for reporting systematic reviews BMJ 2021; 372:n71 doi:10.1136/bmj.n71. Open License, Prisma-Satement.org, material has been adapted).

*Pubmed, ScienceDirect and ResearchGate. **No automation tools were used; all records were excluded by a human”.

There was a mistake in **Figures 2**, **3**, **4** as published. The image used in **Figure 3** should have been **Figure 2** and its caption was moved to **Figure 4**; the image used in **Figure 4** should have been **Figure 3** and its caption was moved to **Figure 2**; the image used in **Figure 2** should have been **Figure 4** and its caption was moved to **Figure 3**. The corrected figures appear below.

**Figure 2:**
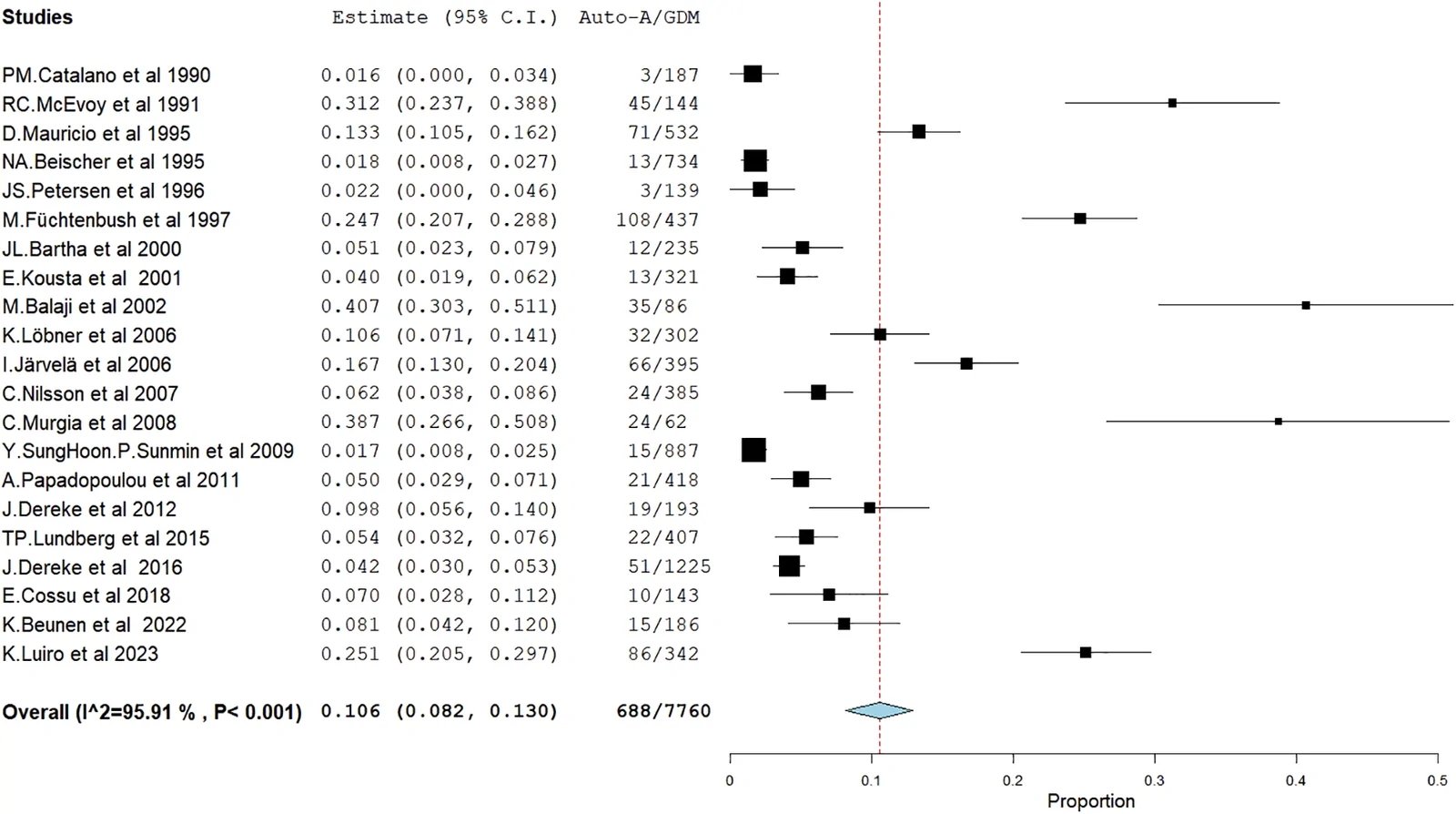
Prevalence of islet autoantibodies positivity in patients with GDM. Forest plot of prevalence of islet autoantibody positivity in pregnant women with GDM is shown. 21 studies including 7760 patients were analyzed (5, 18–37).

**Figure 3:**
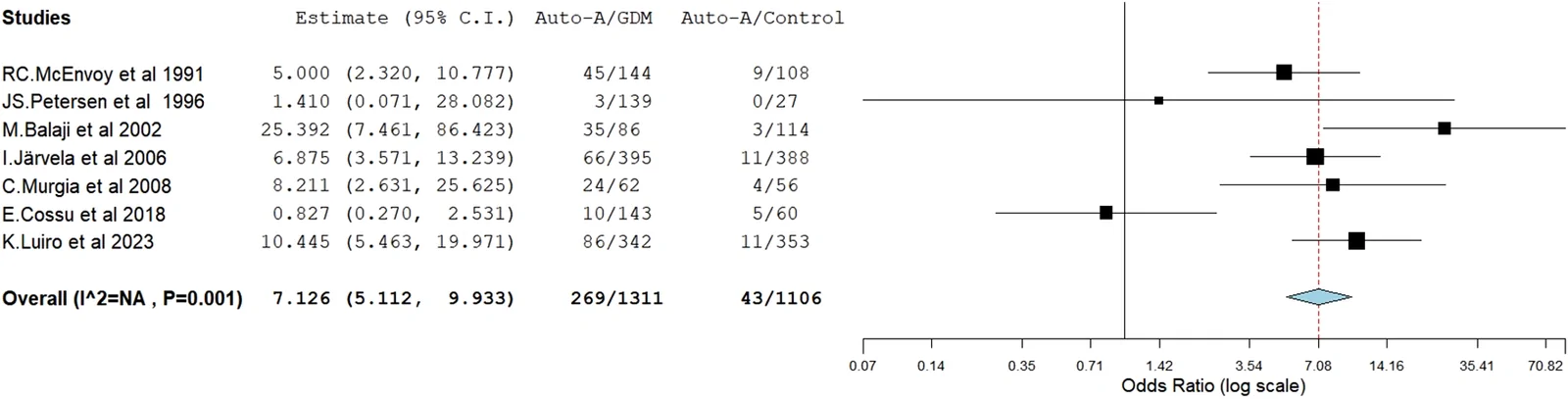
Prevalence of islet autoantibody positivity in pregnant women with and without GDM. Forest plot comparing prevalence of islet autoantibody positivity in patients with GDM (n=1311) and pregnant women without GDM (n=1106) is shown (5, 19–22, 24, 25).

**Figure 4:**
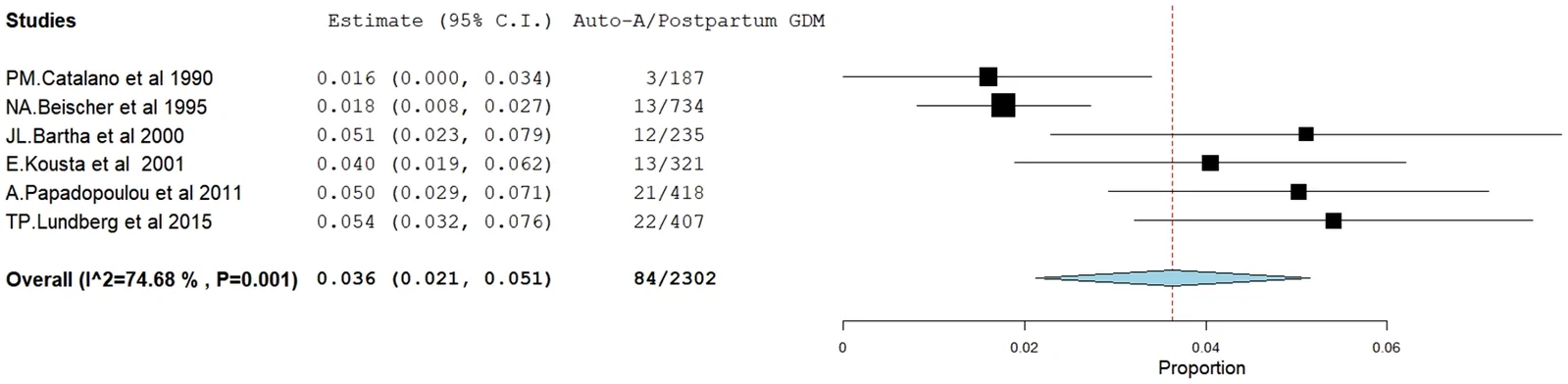
Prevalence of islet autoantibody positivity in the postpartum period. Forest plot of prevalence of islet autoantibody positivity is shown. 6 studies including 2302 patients with GDM were analyzed. Laboratory analyses were performed 1 week to 22 months after delivery (18, 23, 27, 29, 30, 35).

The original version of this article has been updated.

## References

[5] Järvelä IY Juutinen J Koskela P Hartikainen AL Kulmala P Knip M . Gestational diabetes identifies women at risk for permanent type 1 and type 2 diabetes in fertile age: predictive role of autoantibodies. Diabetes Care. (2006) 29:607–12. doi: 10.2337/diacare.29.03.06.dc05-1118 16505514

[18] Catalano PM Tyzbir ED Sims EA . Incidence and significance of islet cell antibodies in women with previous gestational diabetes. Diabetes Care. (1990) 13:478–82. doi: 10.2337/diacare.13.5.478 2190774

[19] McEvoy RC Franklin B Ginsberg-Fellner F . Gestational diabetes mellitus: evidence for autoimmunity against the pancreatic beta cells. Diabetologia. (1991) 34:507–10. doi: 10.1007/BF00403287 1916056

[20] Petersen JS Dyrberg T Damm P Kühl C Mølsted-Pedersen L Buschard K . GAD65 autoantibodies in women with gestational or insulin-dependent diabetes mellitus diagnosed during pregnancy. Diabetologia. (1996) 39:1329–33. doi: 10.1007/s001250050578 8933000

[21] Balaji M Shtauvere-Brameus A Balaji V Seshiah V Sanjeevi CB . Women diagnosed with gestational diabetes mellitus do not carry antibodies against minor islet cell antigens. Ann N Y Acad Sci. (2002) 958:281–4. doi: 10.1111/j.1749-6632.2002.tb02987.x 12021124

[22] Murgia C Orrù M Portoghese E Garau N Zedda P Berria R . Autoimmunity in gestational diabetes mellitus in Sardinia: a preliminary case-control study. Reprod Biol Endocrinol. (2008) 6:24. doi: 10.1186/1477-7827-6-24 18588707 PMC2459179

[23] Papadopoulou A Lynch KF Anderberg E Landin-Olsson M Hansson I Agardh CD . HLA-DQB1 genotypes and islet cell autoantibodies against GAD65 and IA-2 in relation to development of diabetes postpartum in women with gestational diabetes mellitus. Diabetes Res Clin Pract. (2012) 95:260–4. doi: 10.1016/j.diabres.2011.10.037 22104260

[24] Cossu E Incani M Pani MG Gattu G Serafini C Strazzera A . Presence of diabetes-specific autoimmunity in women with gestational diabetes mellitus predicts impaired glucose regulation at follow-up. J Endocrinol Invest. (2018) 41:1061–8. doi: 10.1007/s40618-018-0830-3 29340972

[25] Luiro K Auvinen AM Auvinen J Jokelainen J Järvelä I Knip M . Autoantibodies predict type 1 diabetes after gestational diabetes: a 23-year cohort study. Front Endocrinol (Lausanne). (2023) 14:1286375. doi: 10.3389/fendo.2023.1286375 38192417 PMC10773701

[26] Mauricio D Corcoy R Codina M Morales J Balsells M de Leiva A . Islet cell antibodies and beta-cell function in gestational diabetic women: comparison to firstdegree relatives of type 1 (insulin-dependent) diabetic subjects. Diabetes Med. (1995) 12:1009–14. doi: 10.1111/j.1464-5491.1995.tb00414.x 8582122

[27] Beischer NA Wein P Sheedy MT Mackay IR Rowley MJ Zimmet P . Prevalence of antibodies to glutamic acid decarboxylase in women who have had gestational diabetes. Am J Obstet Gynecol. (1995) 173:1563–9. doi: 10.1016/0002-9378(95)90650-9 7503202

[28] Fuchtenbusch M Ferber K Standl E Ziegler AG . Prediction of type 1 diabetes postpartum in patients with gestational diabetes mellitus by combined islet cell autoantibody screening: a prospective multicenter study. Diabetes. (1997) 46:1459–67. doi: 10.2337/diabetes.46.9.1459 9287047

[29] Bartha JL Martinez-Del-Fresno P Comino-Delgado R . Postpartum metabolism and autoantibody markers in women with gestational diabetes mellitus diagnosed in early pregnancy. Am J Obstet Gynecol. (2001) 184:965–70. doi: 10.1067/mob.2001.112394 11303206

[30] Kousta E Lawrence NJ Anyaoku V Johnston DG McCarthy MI . Prevalence and features of pancreatic islet cell autoimmunity in women with gestational diabetes from different ethnic groups. BJOG. (2001) 108:716–20. doi: 10.1111/j.1471-0528.2001.00180.x 11467697

[31] Löbner K Knopff A Baumgarten A Mollenhauer U Marienfeld S Garrido-Franco M . Predictors of postpartum diabetes in women with gestational diabetes mellitus. Diabetes. (2006) 55:792–7. doi: 10.2337/diabetes.55.03.06.db05-0746 16505245

[32] Nilsson C Ursing D Törn C Åberg A Landin-Olsson M . Presence of GAD antibodies during gestational diabetes mellitus predicts type 1 diabetes. Diabetes Care. (2007) 30:1968–71. doi: 10.2337/dc07-0157 17519433

[33] Yu SH Park S Kim HS Park SY Yim CH Han KO . The prevalence of GAD antibodies in Korean women with gestational diabetesmellitus and their clinical characteristics during and after pregnancy. DiabetesMetab Res Rev. (2009) 25:329–34. doi: 10.1002/dmrr.963 19405080

[34] Dereke J Nilsson C Landin-Olsson M Hillman M . Prevalence of zinc transporter 8 antibodies in gestational diabetes mellitus. Diabetes Med. (2012) 29:e436–39. doi: 10.1111/j.1464-5491.2012.03766.x 22924602

[35] Lundberg TP Højlund K Snogdal LS Jensen DM . Glutamic acid decarboxylase autoantibody positivity postpartum is associated with impaired b-cell function in women with gestational diabetes mellitus. Diabetes Med. (2015) 32:198–206. doi: 10.1111/dme.12615 25345799

[36] Dereke J Nilsson C Strevens H Landin-Olsson M Hillman M . IgG4 subclass glutamic acid decarboxylase antibodies are associated with a reduced risk of developing type 1 diabetes and increased C-peptide levels in GADA-positive gestational diabetes. Clin Immunol. (2016) 162:45–8. doi: 10.1016/j.clim.2015.11.001 26548838

[37] Beunen K Vercauter L Van Crombrugge P Moyson C Verhaeghe J Vandeginste S . Type 1 diabetes-related autoimmune antibodies in women with gestational diabetes mellitus and the long-term risk for glucose intolerance. Front Endocrinol (Lausanne). (2022) 13:973820. doi: 10.3389/fendo.2022.973820 36093103 PMC9449803

