# Supplementary material for: Islet-autoantibodies in gestational diabetes and risk of progression to postpartum diabetes: a systematic review and meta-analysis
Source: Front Endocrinol (Lausanne). 2026 Aug 6;17:1874930. doi: 10.3389/fendo.2026.1874930 (PMC13489886; doi:10.3389/fendo.2026.1874930)
Supplement: Supplementary file 1 [file Table1.pdf]

Supplementary Table 1. Risk-of-bias/quality assessement

Study	Q1	Q2	Q3	Q4	Q5	Q6	Q7	Q8	Q9	Q10	Q11	Q12	Q13	Q14	Overall
Catalano et al., 1990	Y	Y	NR	Y	N	N	N	N	Y	NA	Y	NR	NA	N	Fair
McEvoy et al., 1991	Y	Y	Y	Y	N	N	Y	N	Y	NA	Y	NR	Y	N	Good
Petersen et al., 1996	Y	Y	NR	Y	N	N	Y	N	Y	NA	Y	NR	NA	N	Fair
Balaji et al., 2002	Y	Y	NR	Y	N	N	N	N	Y	NA	Y	NR	NA	N	Fair
Järvelä et al., 2006	Y	Y	Y	Y	N	Y	Y	N	Y	N	Y	NR	Y	Y	Good
Murgia et al., 2008	Y	Y	NR	Y	N	N	Y	N	Y	NA	Y	NR	NA	N	Fair
Papadopoulou et al., 2011	Y	Y	NR	Y	N	Y	Y	N	Y	N	Y	NR	Y	Y	Good
Cossu et al., 2018	Y	Y	NR	Y	N	Y	Y	N	Y	N	Y	NR	Y	Y	Good
Luiro et al., 2023	Y	Y	Y	Y	Y	Y	Y	N	Y	N	Y	NR	Y	Y	Good
Mauricio et al., 1995	Y	Y	NR	Y	N	N	N	N	Y	NA	Y	NR	NA	N	Fair
Beischer et al., 1995	Y	Y	Y	Y	N	N	Y	NA	Y	NA	Y	NA	NR	N	Good
Füchtenbusch et al., 1997	Y	Y	NR	Y	N	Y	Y	N	Y	Y	Y	NR	Y	Y	Good
Bartha et al., 2000	Y	Y	NR	Y	N	Y	Y	N	Y	N	Y	NR	NA	NA	Good
Kousta et al. 2001	Y	Y	NR	Y	N	N	N	N	Y	NA	Y	NR	NA	N	Fair
Löbner et al., 2006	Y	Y	Y	Y	N	Y	Y	Y	Y	Y	Y	NR	Y	NR	Good
Nilsson et al., 2007	Y	Y	Y	Y	N	Y	Y	N	Y	N	Y	NR	Y	Y	Good
Yu et al., 2009	Y	Y	NR	Y	N	Y	Y	N	Y	N	Y	NR	NR	Y	Good
Dereke et al., 2012	Y	Y	NR	Y	N	N	N	N	Y	NA	Y	NR	NA	N	Fair
Lundberg et al., 2015	Y	Y	NR	Y	N	Y	Y	N	Y	N	Y	NR	Y	Y	Good
Dereke et al., 2016	Y	Y	NR	Y	N	Y	Y	N	Y	N	Y	NR	NR	Y	Good
Beunen et al., 2022	Y	Y	Y	Y	Y	NR	Y	N	Y	N	Y	NR	Y	Y	Good

Coding:

Questions (Q)

Q1 Was the research question or objective clearly stated?

Q2 Was the study population clearly specified and defined?

Q3 Was the participation rate of eligible persons at least 50%?

Q4 Were all subjects selected or recruited from similar populations and were inclusion/exclusion criteria prespecified and uniformly applied?

Q5 Was a sample size justification, power description, or variance/effect estimate provided?

Q6 For longitudinal studies, were exposures measured before outcomes?

Q7 Was the timeframe sufficient to reasonably expect an association between exposure and outcome?

Q8 For exposures that can vary over time, did the study examine different levels of exposure?

Q9 Were exposure measures clearly defined, valid, reliable, and consistently implemented?

Q10 Was exposure assessed more than once over time?

Q11 Were outcome measures clearly defined, valid, reliable, and consistently implemented?

Q12 Were outcome assessors blinded to exposure status?

Q13 Was loss to follow-up after baseline 20% or less?

Q14 Were key potential confounding variables measured and statistically adjusted?

Y = Yes, N = No, NR = Not reported / cannot determine from available information, NA = Not applicable
