# Supplementary material for: Islet-autoantibodies in gestational diabetes and risk of progression to postpartum diabetes: a systematic review and meta-analysis
Source: Front Endocrinol (Lausanne). 2026 Aug 6;17:1874930. doi: 10.3389/fendo.2026.1874930 (PMC13489886; doi:10.3389/fendo.2026.1874930)
Supplement: Supplementary file 2 [file Table2.docx]

| Reference | Study population | Time of autoantibody analysis |
| --- | --- | --- |
| Catalano et al | Patients with GDM treated at diabetes center evaluated for participation | Within 1 week of delivery |
| McEvoy et al | Patients with a positive screening test treated at diabetes center evaluated for participation | 18^th^ to 28^th^ gestational week |
| Petersen et al | Patients with diagnosed with GDM treated at diabetes center | 12^th^ to 16^th^ gestational week |
| Balaji et al | Inclusion criteria not reported | Not reported |
| Järvela et al | Patients with GDM and singleton pregnancy treated at diabetes center evaluated for participation | First trimester |
| Murgia et al | Patients with GDM referred to obstetric center evaluated for participation | 17^th^ to 41^st^ gestational week |
| Papadoplu et al | Participants of Mamma study evaluated | 12^nd^ gestational week (positive family history)  28^th^ gestational week  (without family history) |
| Cossu et al | Patients with diagnosed with GDM treated at diabetes center | Not reported |
| Luiro et al | Patients with diagnosed with GDM treated at diabetes center | First trimester |
